# Nifuroxazide Prevents Chikungunya Virus Infection Both *In Vitro* and *In Vivo* via Suppressing Viral Replication

**DOI:** 10.3390/v16081322

**Published:** 2024-08-19

**Authors:** Yangang Liu, Mingxiao Xu, Binghui Xia, Zhuoyue Qiao, Yanhua He, Yan Liu, Zhendong Pan, Congcong Zhang, Haoran Peng, Xuesong Liang, Ping Zhao, Hailin Tang, Xu Zheng

**Affiliations:** 1 Department of Microbiology, Faculty of Naval Medicine, Naval Medical University, Shanghai 200433, China; lyg@smmu.edu.cn (Y.L.); xbhnjucpu@163.com (B.X.); yanhua0556@163.com (Y.H.); 15801952518@163.com (Y.L.); panzhendong1998@126.com (Z.P.); luxion0227@gmail.com (C.Z.); phran@126.com (H.P.); pnzhao@163.com (P.Z.); 2 Key Laboratory of Biological Defense, Ministry of Education, Naval Medical University, Shanghai 200433, China; 3 Department of Infection Diseases, First Affiliated Hospital of Navy Military Medical University, Shanghai 200433, China; 18301997552@163.com (M.X.); liangxuesong2000@163.com (X.L.); 4 Key Laboratory of Chemistry in Ethnic Medicinal Resources, State Ethnic Affairs Commission & Ministry of Education, Yunnan Minzu University, Kunming 650500, China; zhuoy0729@163.com

**Keywords:** Chikungunya virus, arboviruses, nifuroxazide, viral replication, antiviral drug

## Abstract

Chikungunya virus (CHIKV) is a reemerging arbovirus causing disease on a global scale, and the potential for its epidemics remains high. CHIKV has caused millions of cases and heavy economic burdens around the world, while there are no available approved antiviral therapies to date. In this study, nifuroxazide, an FDA-approved antibiotic for acute diarrhea or colitis, was found to significantly inhibit a variety of arboviruses, although its antiviral activity varied among different target cell types. Nifuroxazide exhibited relatively high inhibitory efficiency in yellow fever virus (YFV) infection of the hepatoma cell line Huh7, tick-borne encephalitis virus (TBEV) and west nile virus (WNV) infection of the vascular endothelial cell line HUVEC, and CHIKV infection of both Huh7 cells and HUVECs, while it barely affected the viral invasion of neurons. Further systematic studies on the action stage of nifuroxazide showed that nifuroxazide mainly inhibited in the viral replication stage. *In vivo*, nifuroxazide significantly reduced the viral load in muscles and protected mice from CHIKV-induced footpad swelling, an inflammation injury within the arthrosis of infected mice. These results suggest that nifuroxazide has a potential clinical application as an antiviral drug, such as in the treatment of CHIKV infection.

## 1. Introduction

Chikungunya virus (CHIKV) is a mosquito-borne virus responsible for periodic and explosive outbreaks of a febrile disease that is characterized by severe and sometimes prolonged polyarthritis [1]. Since the first reports of CHIKV infection in Africa in the 1950s, subsequent epidemics of CHIKV occurred throughout the latter half of the 20th century in countries within Asia and sub-Saharan Africa [2]. Since 2006, it has spread to new areas, causing disease on a global scale, and the potential for CHIKV epidemics remains high [3,4]. The transmission of CHIKV was vectored mainly by *Aedes aegypti* and *Aede albopictus* mosquitoes [5]. After deposition into the bloodstream or skin through the bite of an infected mosquito, CHIKV replicates at the site of inoculation in fibroblasts and possibly macrophages [1,6]. Despite triggering innate immune responses, the virus spreads via lymphatics into the bloodstream, allowing dissemination to several sites of replication, most commonly lymphoid organs (lymph nodes and spleen), skin, and especially tissues where prominent disease symptoms occur (muscle, peripheral joints, and tendons), but also in the brain and liver in more severe cases [7,8,9]. CHIKV disease is often self-limiting and has a low fatality rate, but it has a high fatality rate and disability rate due to CHIKV-associated central nervous system (CNS) disease in infants and elderly individuals [10,11]. However, no approved drugs are available for the specific treatment of CHIKV to date.

Drug repurposing is an important strategy for the discovery of clinical candidates for emerging viruses [12,13,14,15]. As a safe nitrofuran antibacterial drug used clinically to treat acute diarrhea and infectious traveler diarrhea or colitis, nifuroxazide is a potential effective anticancer drug for various types of cancer and a therapeutic candidate for the treatment of schistosomiasis [16,17]. Currently, there are no reported antiviral activities of nifuroxazide, and there are a variety of anticancer and antibacterial drugs exhibiting solid antiviral activity [18,19]. In this study, FDA-approved nifuroxazide was repurposed for the assessment of antiviral ability on CHIKV and other arboviruses such as west nile virus (WNV), yellow fever virus (YFV), and tick-borne encephalitis virus (TBEV). Nifuroxazide exerted high antiviral potency against CHIKV, WNV, YFV, and TBEV by inhibiting viral replication *in vitro*, showing low-micromolar antiviral effects. Moreover, nifuroxazide also displayed potent anti-CHIKV activity *in vivo*. To our knowledge, this research is the first to describe the antiviral efficacy of nifuroxazide against reemerging flaviviruses and alphaviruses, and it demonstrates that nifuroxazide is a promising broad-spectrum antiviral candidate.

## 2. Materials and Methods

### 2.1. Cell Lines, Viruses, and Compounds

SH-SY5Y (CRL-2266, ATCC, MA, USA), Huh7 (SCSP-526, Chinese Academy of Sciences, Shanghai, China), and Vero (CCL-81, ATCC) cell lines were cultured in Dulbecco’s Modified Eagle’s medium (DMEM, Thermo Fisher Scientific, CA, USA). HUVECs (CRL-1730, ATCC) were cultured in an endothelial cell medium (Sciencell, CA, USA) containing an endothelial cell growth factor. All cells were incubated in a humidified incubator at 37 °C in 5% CO_2_. Nifuroxazide (10 mM) was purchased from Selleck Chemicals (Houston, TX, USA). CHIKV strain LR2006 OPY1 (DQ443544), TBEV strain Zmeinogorsk-5 (KY069125), and WNV strain NY2000 (AF404756) were synthesized in this laboratory [20]. The YFV strain FJYF03/2016 (KY587416) was isolated from the serum of a confirmed yellow fever patient [21]. All mouse experiments were conducted under biosafety level 3 conditions according to the *Guide for Animal Care and Use* (Naval Medical University, Shanghai, China) and were approved by the Animal Care and Use Committee (license number: NMU-20230310-16).

### 2.2. Drug Inhibition Assay

Nifuroxazide or dimethyl sulfoxide (DMSO, control) were diluted in DMEM (2% FBS) to reach the indicated concentrations. For dose-response, binding, and endocytosis assays, drugs were 2-fold serially diluted to generate a working solution with concentrations ranging from 1.625 μM to 50 μM. Cells were grown on 96-well plates to reach 90% confluence and incubated with the virus at a multiplicity of infection (MOI) of 0.1 in the infection medium (DMEM, 1% penicillin/streptomycin) at 37 °C for 2 h. Then, they were treated with drugs for another 22 h before being lysed by TRIzol (TaKaRa, Shiga, Japan) or fixed with methyl alcohol for further real-time quantitative PCR (RT-qPCR) or immunofluorescence (IF) analysis (see Supplementary Materials). In parallel, uninfected cells were incubated with the same drugs for 22 h, and the cell viability was assessed by a CCK-8 kit (Beyotime, Suzhou, China). The half inhibitory concentration (IC50) and half cytotoxic concentration (CC50) of nifuroxazide was generated by GraphPad Prism 10.3 (GraphPad Software, San Diego, CA, USA) using nonlinear regression models. The selective index (SI) of nifuroxazide was further calculated by dividing CC50 by IC50 (Table S1).

### 2.3. Immunofluorescence (IF) Assay

The infectivity of a virus was determined by an immunofluorescence assay as previously described [22]. Simply, cells were fixed in methanol at −20 °C for 20 min, blocked with 3% BSA for 2 h at room temperature (RT), and incubated with antibodies against CHIKV, YFV, WNV, or TBEV at 4 °C overnight. Then, the cells were washed twice in PBS and incubated with an AF-488-conjugated second antibody (Thermo Fisher Scientific, CA, USA) for 1 h at RT in the dark. After being washed, the cells were then counterstained with DAPI (Sigma-Aldrich, MO, USA) for 15 min at RT in the dark. The plates were scanned, and the infected or total cells were counted with Cytation 5 (Biotek Instruments, CA, USA). The percentage of infected cells (infectivity) was calculated and normalized to the control group. Information regarding the antibodies used for immunofluorescence is provided in Table S2.

### 2.4. Cell Viability Assay

To assess the cytotoxicity of nifuroxazide, a Cell Counting Kit-8 (CCK-8) (Beyotime, Suzhou, China) was used according to the manufacturer’s instructions. Briefly, cells grown on 96-well plates were treated by nifuroxazide or DMSO after confluence for 22 h. After being washed twice with PBS, the cells were incubated with a diluted CCK-8 solution (1:9) at 37 °C for 4 h. Finally, the plates were read by a microplate reader (BioTek, Vermont, VT, USA), and the cell viability was calculated and normalized to the DMSO-treated group.

### 2.5. Real-Time Quantitative PCR (RT-qPCR)

Cells or tissues were first lysed using TRIzol (TaKaRa, Shiga, Japan), and the total RNA was extracted using standard methods. The total RNA was then used as the template to synthesize cDNA using a PrimeScript RT Master Mix kit (TaKaRa) following the manufacturer’s instructions. Finally, PCR amplification of the target genes was performed using a SYBR Premix Ex Taq kit (TaKaRa) in an ABI 7300 system (Applied Biosystems, Waltham, MA, USA). The Glyceraldehyde-3-Phosphate Dehydrogenase (GAPDH) was taken as an internal reference gene. Primer pairs used in this study are illustrated in Table S3. The relative quantitation (RQ) of gene expression was calculated using comparative cycle threshold values (2-ΔΔCt), as exhibited below (1):

RQ_target_ = 2 ^- (drug[CT*target* – CT*GAPDH*] - blank[CT*target*– CT*GAPDH*])^ (1)

### 2.6. Viral Kinetics Assay

To determine the replication and releasing kinetics, Huh7 cells with 90% confluence were first bound with CHIKV (MOI = 0.1) at 4 °C for 1 h. The cells were washed twice with PBS and transferred to 37 °C. At different time points, the cells and supernatants were collected, and the total RNA was isolated using TRIzol (TAKARA) or TRIzol LS (Thermo Fisher Scientific), respectively, and subjected to RT-qPCR analysis for CHIKV RNA.

### 2.7. Time-of-Drug-Addition Assay

The time-of-drug-addition assay was carried out as reported [21]. Briefly, after reaching 90% confluence in 96-well plates, Huh7 cells were incubated with CHIKV (MOI = 0.1) for 2 h. During different periods of infection (−2–0, 0–2, 2–4, 4–6, 6–8, 8–10, 10–12, 12–14, and 14–24 h), the supernatants were replaced with a fresh medium containing nifuroxazide (25 μM) or DMSO and co-cultured for 2 h. The −2–0 h time range means the 2 h period before infection, and the 14–24 h means the latest period of infection. The schematic diagram of drug addition was described in [20]. At 24 h post-infection (hpi), the cells were washed twice with PBS, fixed, and subjected to an IF test.

### 2.8. Binding Assay

Huh7 cells were grown on 24-well plates to reach 100% confluence and incubated with CHIKV (MOI = 1) at 4 °C for 2 h together with different concentrations of nifuroxazide or DMSO. Then, the plates were washed twice with PBS and either lysed immediately with TRIzol for RT-qPCR analysis of bound CHIKV or further cultured in a fresh medium for IF assay at 24 h post-temperature shift.

### 2.9. Endocytosis Assay

An endocytosis assay was performed as described [23]. In brief, after reaching 100% confluence, Huh7 cells were incubated with CHIKV (MOI = 1) at 4 °C for 1 h. Then, the plates were washed twice with PBS, supplied with a culture medium containing different concentrations of nifuroxazide, and transferred to 37 °C for viral entry. Two hours after being transferred to 37 °C, the cells were washed twice and incubated with proteinase K (PK, 1 mg/mL in PBS) (Thermo Fisher Scientific) or PBS (negative control) for 30 min at 4 °C to remove un-internalized virions. Next, the cells were gently washed twice with PBS, lysed by TRIzol, and subjected to RT-qPCR analysis. The relative quantity (RQ) of the internalized virus is expressed as follows (2):

RQ_endocytosis_ = 2^-ΔΔCt-proteinaseK^ − (2^-ΔΔCt-PBS^ − 2^-ΔΔCt-Binding^) (2)

### 2.10. Transferrin Uptake Assay

A transferrin uptake assay was carried out according to a previous study [23]. Simply, Huh7 cells were seeded in 8-well chamber slides (5 × 10^3^ cells/well) and cultured overnight before incubating with AF-488-conjugated transferrin (AF-transferrin, Molecular Probes, MN, USA) for 2 h at 37 °C. After being washed once with wash buffer (150 mM NaCl, 0.1 M glycine, pH 2.5) and three times with PBS, the cells were fixed with cold methanol (−20 °C, 20 min) and viewed by a Zeiss LSM 710 confocal microscope.

### 2.11. Membrane Fusion Assay

Viral-cell membrane fusion was assessed by labeling CHIKV with a self-quenching concentration of a fluorescent lipid, DiD, as reported [24]. To generate DiD-labelled CHIKV (CHIKV^DiD^), 5 μL of Vybrant^®^ DiD cell-labelling solution (1 mM) (Invitrogen, Carlsbad, CA) was mixed with 1 mL of virus stock (approximately 10^8^ PFU) under vertexing and incubated at 37 °C for 30 min. The mixture was then loaded on a 20% sucrose cushion and separated by 4 h of ultracentrifugation (10,000× *g*, 4 °C). The precipitation was further washed twice with PBS and resuspended in DMEM with 2% FBS. Huh7 cells were incubated with the purified CHIKV^DiD^ at 37 °C together with nifuroxazide (25 μM), NH_4_Cl (25 mM), bafilomycin A1 (25 nM), DMSO, or PBS (blank). The plates were immediately read by a Microplate Reader (Biotek Instruments, CA, USA) (644 nm excitation/665 nm emission) for the DiD dequenching every 10 min for a total of 48 cycles. A cell-free group (with CHIKV^DiD^) was used as a background control. The relative fluorescence unit (RFU) of drugs is expressed as follows (3):

RFU_drug_ = FU_drug_ − FU_background_ (3)

### 2.12. Genome Transfection Assay

A genome transfection assay was performed as previously described to assess viral replication, assembly, and release [21]. Briefly, the genome RNA of CHIKV was isolated from virus stock using a QIAamp Viral RNA Mini Kit (Qiagen, Germany) and transfected into 100% confluence of Huh7 cells using Lipofectamin^TM^ 2000 (Lipo2000) according to the manufacturer’s instructions. Six hours later, the supernatants were replaced with a working drug solution and cultured for another 6 h (for the replication test) or 18 h (for the assembly and release test). For the replication test, the cells and supernatants were collected and the total RNA was extracted using TRIzol or TRIzol LS, respectively, and subjected to RT-qPCR analysis for CHIKV RNA. The replication level of CHIKV was represented by the total RNA in the cells and supernatants. For the assembly and release test, besides the analysis of total RNA, infectious viruses in the supernatants (released, directly collected) and the cells (assembled, collected by three cycles of freeze and thaw in liquid nitrogen) were collected. Then, the infectivity of both the released and assembled virions were assessed by an IF assay. The assembly (AS) or release (RE) ratios were expressed as follows and normalized to the DMSO group (4):

AS/RE ratio = (AS/RE Infectivity_drug_)/(Total RNA_drug_) (4)

### 2.13. Replicon Expression Assay

The replicon plasmids of the CHIKV strain LR2006-OPY1 (carrying EGFP and the NanoLuc reporter gene) and strain YFV-17D (carrying the NanoLuc reporter gene) were synthesized as described [20,22]. The schematic diagram of the replicon plasmids is shown in Figure S1. The plasmids were then linearized by *SmaI* (for the YFV-17D replicon) and *NotI* (for the CHIKV-LR2006 replicon) and subjected to transcription assay using a HiScribe T7 ARCA mRNA Kit (New England Biolabs, USA). The viral replicon RNAs were then transfected into cells by Lipo2000. Six hours after transfection, the cells were treated with different doses of nifuroxazide or DMSO for another 18 h. The expression of the reporter gene, NanoLuc, was evaluated by adding 10% NanoLuc reagent, incubating for 5 min in the dark, and scanning using a Chemiluminescence Imaging System (Clinx Science instruments, Shanghai, China). The expression of EGFP was captured using Cytation 5. The gray intensity of NanoLuc and the mean fluorescence intensity of EGFP results were analyzed by ImageJ and Gen5 3.10, respectively, and normalized to the DMSO group.

### 2.14. In Vivo Experiment

Four-week-old C57BL/6 mice were inoculated subcutaneously (s.c.) in the left rear footpad with 1000 PFU of CHIKV-LR2006 or PBS (mock) followed by daily oral administration (p.o.) of nifuroxazide or DMSO in corn oil. They were given a nifuroxazide safe dose at 50 mg/kg/days, as described in [25], from the day before infection to the third day post infection (−1–3 dpi). Mice were monitored for foot swelling using calipers daily for 10 days. At 2, 4, 6, and 8 dpi, mice were euthanized and the left (ipsilateral) and right (contralateral) rear feet were obtained. For histological analysis, the tissues were embedded in paraffin, prepared in 5 μm sections, stained with hematoxylin and eosin (HE), and imaged using Cytation 5. For viral load evaluation, tissues were harvested in PBS, homogenized in TRIzol, and analyzed with RT-qPCR or ground in PBS for determination of infectious virions via plaque assay [21,26].

### 2.15. Statistical Analysis

All data were analyzed in GraphPad Prism 10.3 (GraphPad Software, CA, USA). Student’s t-test was applied to evaluate the statistical difference between two unpaired groups. One-way ANOVA was used to determine the statistical significance between multiple groups, and a Bonferroni post-hoc test was used to correct for multiple comparisons. The symbols *, **, and *** in the figures indicate *p* < 0.05, *p* < 0.01, and *p* < 0.001, respectively.

## 3. Results

### 3.1. Nifuroxazide Dose-Dependently Inhibits CHIKV Infection in Huh7 Cells and HUVECs but Not in SY5Y Cells

**Figure 1:**
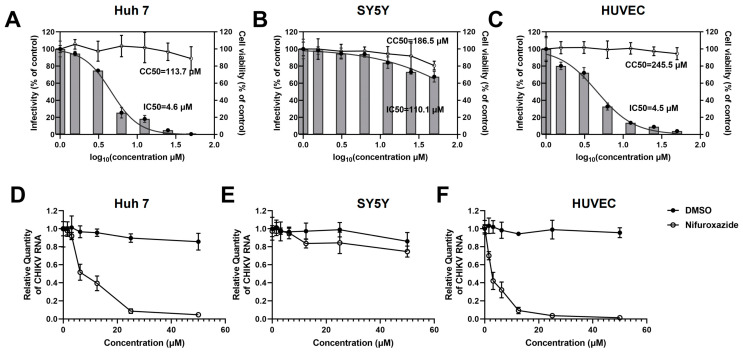
Nifuroxazide suppressing infection of CHIKV in Huh7 cells and HUVECs in a dose-dependent manner. After infected by CHIKV (MOI = 0.1) for 2 h, Huh7 cells (**A**,**D**), SH-SY5Y cells (SY5Y, **B**,**E**), and HUVECs (**C**,**F**) were incubated with different concentrations of nifuroxazide or DMSO. After 24 h of infection: (**A**–**C**) Immunofluorescence (IF) assays were performed at 24 h post infection (24 hpi) and the infectivity was assessed and normalized to 0 μM group (control). Normalized infectivity of nifuroxazide-treated groups were shown as histograms. IC50 values were calculated by GraphPad software using a nonlinear regression model (smooth curves). The bars at 0 of *X* axis represent the control groups; (**A**–**C**, right *Y* axis) CCK-8 kits were utilized to evaluate the cell viability and normalized to control group (broken curves). The CC50 values were calculated by GraphPad software using a nonlinear regression model; (n = 6) (**D**–**F**) RT-qPCR was performed to detect viral RNA load in the cell, and the relative quantity of CHIKV RNA genome was calculated using comparative cycle threshold values (2^-ΔΔCt^). (n = 6).

To investigate the potential of FDA-approved nifuroxazide for repurposing as an antiviral strategy, infection models of human-derived cell lines were utilized to assess the action of nifuroxazide on CHIKV infection *in vitro*. Huh7 cells, SH-SY5Y (SY5Y) cells, and primary human umbilical vein endothelial cells (HUVECs) were exposed to CHIKV (MOI = 0.1) for 2 h, followed by treatment with different doses (six 2-fold serial dilutions, 50 μM to 1.625 μM) of nifuroxazide. Viral infectivity and cell toxicity were determined by immunofluorescence (IF), real-time quantitative PCR (RT-qPCR) assays, and Cell Counting Kit-8 (CCK-8) at 24 hpi. As shown in Figure 1A–C, nifuroxazide significantly suppressed the infectivity of CHIKV in Huh7 (IC50 of 4.6 μM) and HUVECs (IC50 of 4.5 μM) in a dose-dependent manner, while only mildly affecting the infection in SY5Y cells (IC50 of 110.1 μM). Taking the cytotoxicity into account, the selective indexes (SIs) of nifuroxazide on CHIKV infection in Huh7 cells, SY5Y cells, and HUVECs were 24.7, 1.7, and 54.6 (Table S1), respectively, indicating its good therapeutic effect on the infection of liver and vascular endothelial cells but not on neurons. Those results were further confirmed by the analysis of viral RNA (Figure 1D–F), in which the viral load in Huh7 cells and HUVECs, but not in SY5Y cells, were markedly lessened under nifuroxazide treatment in a dose-dependent pattern. Of note, nifuroxazide showed no obvious toxicity to all the cells except SY5Y, on which 50 μM of nifuroxazide induced a viability decrease of about 19.4% (*p* < 0.05).

### 3.2. The Antiviral Efficacy of Nifuroxazide in Other Arboviruses

**Figure 2:**
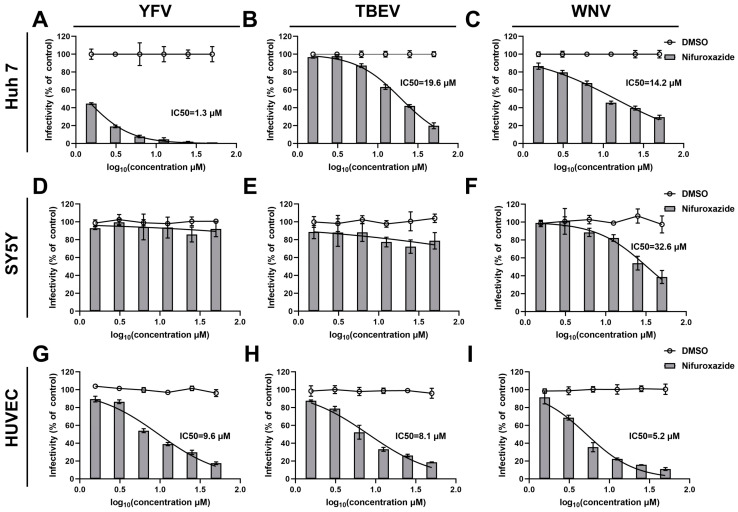
Antiviral efficiency of nifuroxazide on YFV, TBEV, and CHIKV. After incubated with YFV (MOI = 0.1) (**A**,**D**,**G**), TBEV (MOI = 0.1) (**B**,**E**,**H**), and WNV (MOI = 0.1) (**C**,**F**,**I**) for 2 h, Huh7 cells (**A**–**C**), SY5Y cells (**D**–**F**), and HUVECs (**G**–**I**) were treated by different concentrations of nifuroxazide or DMSO. IF assays were conducted at 24 h post infection (24 hpi) to assess viral infectivity. IC50 values were calculated by GraphPad software using a nonlinear regression model (smooth curves). Normalized infectivity of nifuroxazide- and DMSO-treated groups were shown as histograms and broken curves, respectively (n = 6). The broken line refers to the infectivity under different concentrations of DMSO.

To clarify the action of nifuroxazide on other arboviruses, dose-response assays on TBEV, WNV, and YFV were then conducted. Interestingly, as shown in Figure 2D–F, nifuroxazide hardly affected the infection of the three types of viruses on SY5Y cells, though high doses of the drug displayed mild inhibition on WNV invasion of SY5Y cells (IC50 of 32.6 μM). However, infections of all the viruses on Huh7 cells and HUVECs were dose-reliantly suppressed by nifuroxazide, with IC50 values ranging from approximately 1.3 to 19.6 μM (Figure 2A–C,G–I, and Table S1). Among them, nifuroxazide displayed the best therapeutic effect on YFV infection in Huh7, as the selective index (SI) (87.46) exceeded all of the others (Table S1), suggesting an important potential of nifuroxazide in YFV treatment, whose main targeted organ is the liver. In comparison, the efficiency of nifuroxazide on TBEV and WNV infection in Huh7 is much lower, with IC50 values of 19.6 and 14.2 μM, and SIs of 5.80 and 8.01, respectively (Figure 2A,B, and Table S1). On the contrary, in the HUVEC infection model, the antiviral capacity of nifuroxazide on TBEV (IC50 of 8.1 μM and SI of 30.3) and WNV (IC50 of 5.2 μM and SI of 47.2), two neuroinvasive flaviviruses, is higher than that of YFV (IC50 of 9.6 μM and SI of 25.6) (Figure 2G–I, and Table S1). Taken together, nifuroxazide shows potential for repurposing as a candidate to prevent liver damage from YFV and blood brain barrier (BBB) crossing of TBEV and WNV, while its effect on treating the neuropathogenicity of arboviruses may be limited.

### 3.3. Nifuroxazide Shows Minor Effects on the Entry Process of CHIKV

**Figure 3:**
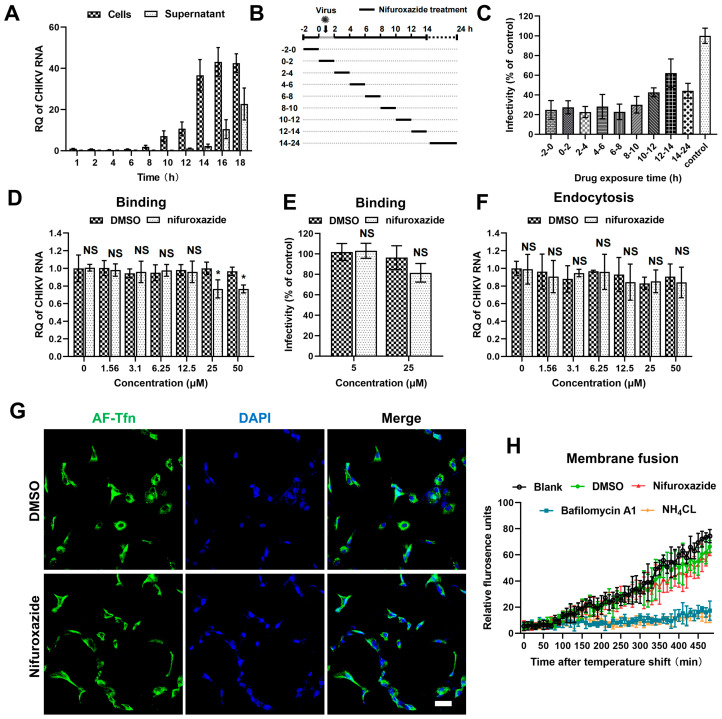
The antiviral effect of nifuroxazide is independent of entry steps. (**A**) Viral kinetics assay: Huh7 cells were incubated with CHIKV (MOI = 0.1) at 4 °C for 1 h, washed, and transferred to a 37 °C culture. Viral RNA in cells or supernatants were isolated at different time points and subjected to RT-qPCR analysis. (n = 3) (**B**) Schematic diagram of time-of-drug-addition assay: Briefly, nifuroxazide (25 μM) or DMSO was added to Huh7 cells during different periods of CHIKV (MOI = 0.1) infection and treated for 2 h, followed by IF assay to detect viral infectivity. (**C**) Result of time-of–drug-addition assay: Relative infectivity was calculated by normalizing to DMSO-treated control. (n = 3) (**D**,**E**) After incubated with CHIKV (MOI = 1) and different doses of nifuroxazide at 4 °C for 2 h, Huh7 cells were washed with PBS buffer, lysed immediately for binding analysis using RT-qPCR (D), or transferred to 37 °C culture for infectivity detection at 24 hpi (**E**). (n = 6) (**F**) Endocytosis assay: After incubated with CHIKV (MOI = 1) at 4 °C for 1 h, Huh7 cells were washed with PBS and further incubated with different doses of nifuroxazide at 37 °C for 2 h. The cells were washed and incubated with proteinase K in PBS and lysed using TRIzol for RT-qPCR analysis. (n = 6) (**G**) Huh7 cells were incubated with AF-488 transferrin (AF-Tfn) and nifuroxazide (25 μM) or DMSO control for 20 min before being fixed and imaged with confocal microscopy. Scale bar, 50 μm. (n = 3) (**H**) CHIKV labeled by DiD (CHIKV^DiD^) was added to cells with nifuroxazide (25 μM), NH_4_Cl (25 mM), bafilomycin A1 (25 nM), DMSO, or PBS (blank), and the fluorescence was monitored every 10 min for 8 h. (n = 3) * *p* < 0.05 compared to DMSO control. NS: not significant.

To uncover the action stage of nifuroxazide in the CHIKV life cycle, the kinetics of viral amplification and release were determined first. RT-qPCR analysis of the viral RNA in cell and culture mediums at different time points post infection showed that the replication of viral genomes was detectable at 8 h post infection (8 hpi) and grew to reach a plateau after 14 hpi, at which point the released viral RNA in the supernatants began to be detectable (Figure 3A). According to this, a time-of–drug-addition assay was then established: CHIKV (MOI = 0.1)-infected Huh7 cells were treated with 2 h of nifuroxazide (25 μM) at different periods of the viral life cycle (Figure 3B). Infectivity determined at 24 hpi showed that nifuroxazide exhibited prominent suppressive activity during all therapeutic windows, especially the 2–10 hpi period, during which the inhibitory efficiency exceeded 70% (Figure 3C), revealing an irreversible or multi-stage action of nifuroxazide.

The cell entry step of a virus is composed of binding, internalizing (endocytosis), and membrane fusion processes [27]. Thus, we next investigated the action of nifuroxazide in CHIKV binding using binding assays and IF assays. As shown in Figure 3D, binding viral RNA was slightly reduced in 25 μM and 50 μM of nifuroxazide (*p* < 0.05). Though decrease of infectivity under treatment of 25 μM of nifuroxazide was also observed in the result of IF, the statistical difference was not significant (*p* > 0.05) (Figure 3E). This suggests that RT-qPCR may be more sensitive than IF in viral binding evaluation, and nifuroxazide only mildly impeded CHIKV’s attachment to the cell surface. Then, endocytosis assays were performed to explore the role of nifuroxazide on the internalization step of CHIKV entry. As illustrated in Figure 3F, the internalization of CHIKV resisted all concentrations of nifuroxazide. In addition, Alexa Fluor-488-conjugated transferrin (AF-Tfn), a cargo of clathrin-mediated endocytosis (CME), was utilized to assess the effect of nifuroxazide on CME, which is reported to be the classic endocytic route of CHIKV [27]. Consistent with the endocytosis assay, no obvious decline was observed in AF-Tfn uptake under the treatment of nifuroxazide, which further confirmed that the antiviral activity of nifuroxazide is independent of the endocytosis stage of CHIKV.

To dynamically monitor the virus-cell membrane fusion, the CHIKV membrane was labelled with DiD, a lipophilic dye. Due to the self-quenching characteristics, the fluorescence signal is undetectable when DiD dye is incorporated into the membrane of a virus at a high concentration. The hydrophobic fluorophore would diffuse into the host membrane, de-quench, and emit fluorescence when the membrane fusion occurred between a virus and host endosome. Based on this, DiD-labelled CHIKV (CHIKV^DiD^) was incubated with Huh7 for membrane fusion determination. We found that membrane fusion-induced fluorescence becomes detectable approximately after 100 min of incubation and continues to increase in untreated (the black line) or DMSO-treated (the green line) cells (Figure 3H). This increasing fluorescence was blocked by treatment of the two inhibitors of endosomal acidification, bafilomycin A1 (the blue line) and NH_4_Cl (the orange line) (Figure 3H). In contrast, treatment of nifuroxazide did not impair the fluorescence signal, meaning there was no action on virus-cell membrane fusion (Figure 3H, the red line). In conclusion, the anti-CHIKV ability of nifuroxazide is independent of the entry stage.

### 3.4. Nifuroxazide Suppressed CHIKV Replication but Not Assembly and Release

**Figure 4:**
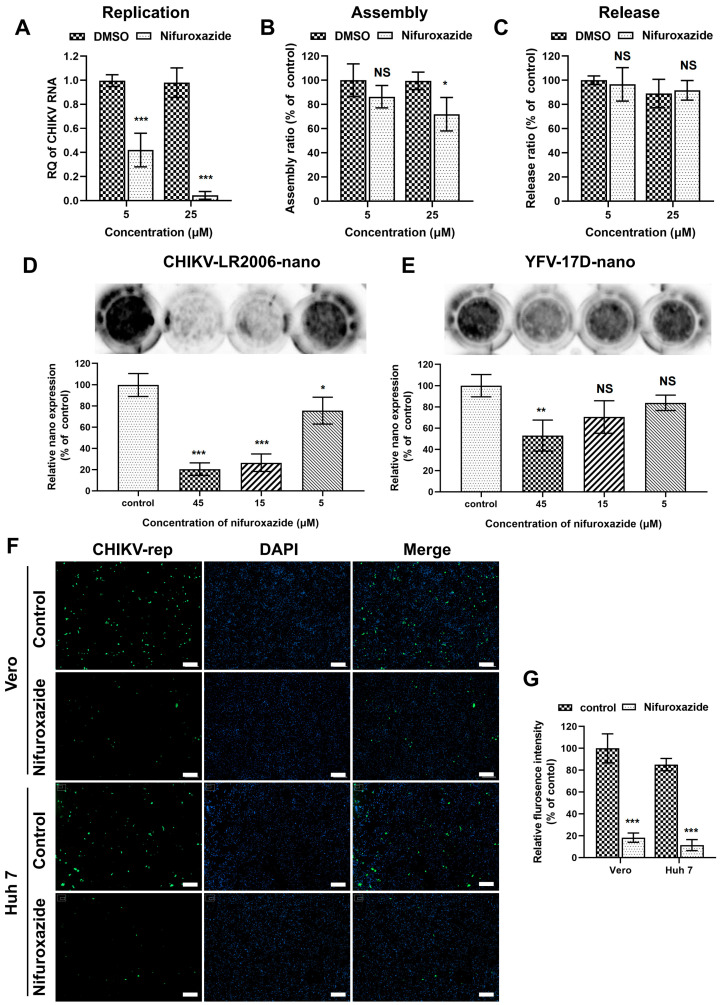
Nifuroxazide inhibits viral replication. (**A**–**C**) Genome transfection assay: after transfected with CHIKV genome for 6 h, Huh7 cells were treated with nifuroxazide (5 μM and 25 μM) for another 6 h (**A**) or 18 h (**B**,**C**). For replication test (A), the RNA from cells and supernatants were extracted and analyzed by RT-qPCR for total viral RNA. For assembly (**B**) and release (**C**) test, assembled (collected by three cycles of freeze and thaw of cells) and released virions were collected and used to infect Huh7 cells. Assembly and release ratios (see Methods and Materials section) were calculated and normalized to DMSO control. (n = 6) (**D**,**E**) Huh7 cells were transfected with CHIKV (CHIKV-LR2006-nano) or YFV (YFV-17D-nano) replicon RNA carrying NanoLuc for 6 h and treated with nifuroxazide (5 μM, 15 μM and 45 μM) or DMSO for another 18 h. The expression of reporter gene was evaluated by adding NanoLuc reagent and scanning with an ECL system, followed by analysis of gray intensity. The expression level of NanoLuc was normalized to DMSO control. (n = 3) (**F**,**G**) Vero or Huh7 cells were transfected with CHIKV-LR2006-replicon (CHIKV-rep) RNA carrying EGFP for 6 h and treated with nifuroxazide (25 μM) or DMSO. Eighteen hours after drug treatment, the EGFP fluorescence was captured (**F**), and mean fluorescence intensity was calculated and normalized to DMSO groups. (n = 6) Scale bar, 200 μm. * *p* < 0.05; ** *p* < 0.01; *** *p* < 0.001 compared to DMSO control. NS: not significant.

After membrane fusion, a viral genome was released into the cytoplasm and initiated post-entry stages, including replication, assembly, and release [28,29]. Since the binding of CHIKV was mildly lessened under nifuroxazide treatment, genome transfection assays were then established to explore the action of nifuroxazide on those stages. To bypass the entry process, Huh7 cells were transfected with genome RNA of CHIKV isolated from virions for 6 h and incubated with nifuroxazide (5 μM and 25 μM) for another 6 h. The total viral RNA in the cells and supernatants, which represented the replication level, was analyzed by RT-qPCR. As shown in Figure 4A, the CHIKV RNA amplification was diminished under treatment of both concentrations of nifuroxazide, especially the 25 μM group in which the viral replication was nearly blocked. For the determination of assembly and release, the time of drug treatment was prolonged to 18 h in the genome transfection assay, and the infectious virions in cells (assembled) and supernatants (released) were respectively collected and quantified by IF assay. The results showed that the assembly ratio decreased a few under treatment of 25 μM, but not 5 μM, of nifuroxazide (*p* < 0.05) (Figure 4B), while the ratio of release exhibited no significant change under either concentration of nifuroxazide (Figure 4C). These results revealed that nifuroxazide works primarily in the stage of CHIKV replication.

To further verify the ability of nifuroxazide to prevent virus replication, single-round CHIKV-LR2006 and YFV-17D replicon plasmids carrying reporter genes were constructed. Firstly, the replicon RNAs carrying nano-luciferase (NanoLuc) transcribed from the linearized plasmids were transfected into Huh7 cells and treated with different doses of nifuroxazide, and the expression of NanoLuc was detected 24 h post transfection (24 hpt). As presented in Figure 4D, nifuroxazide dose-dependently suppresses CHIKV-LR2006-nano expression, and 15 μM and 45 μM of nifuroxazide even showed inhibitory efficiency of more than 70% (*p* < 0.001). However, unlike the highly efficient action in infectivity shown in Figure 2A, only 45 μM of nifuroxazide showed an inhibition on the expression of YFV-17D-nano, with an efficiency of approximately 47.1% (*p* < 0.001) (Figure 4E). Then, a single-round CHIKV-LR2006 replicon carrying enhanced green fluorescent protein (EGFP) was used to further prove the role of nifuroxazide in CHIKV replication. As expected, nifuroxazide (25 μM) could markedly prevent the EGFP fluorescence expressed by the CHIKV-LR2006 replicon in both Huh7 cells (inhibitory efficiency approximately 81.7%, *p* < 0.001) and Vero cells (inhibitory efficiency approximately 73.7%, *p* < 0.001). These results further elucidated the inhibitory effect of nifuroxazide on CHIKV replication.

### 3.5. Nifuroxazide Protects Mice from CHIKV-Induced Severe Disease

**Figure 5:**
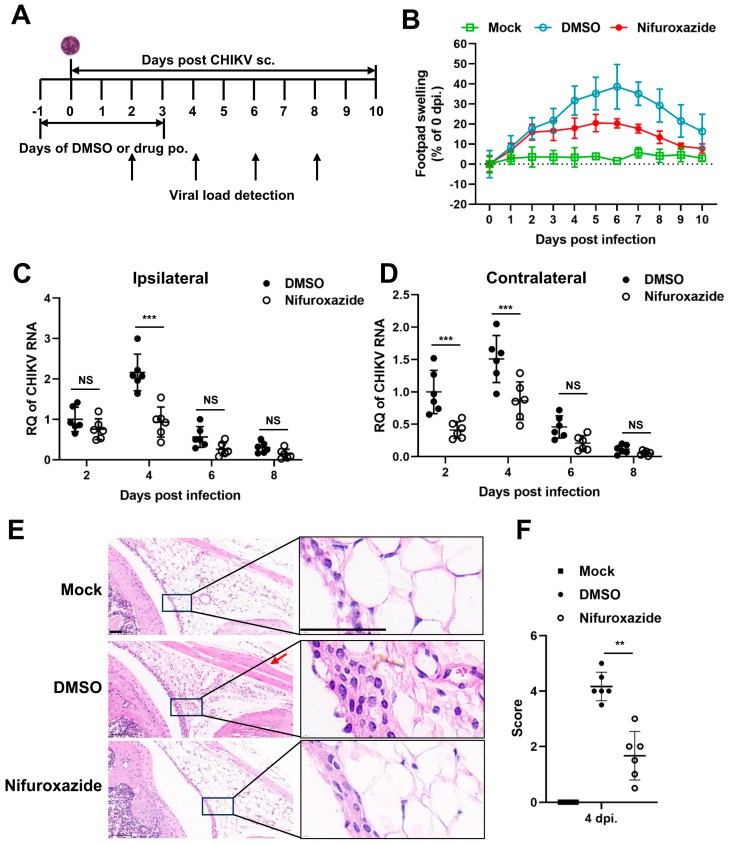
Nifuroxazide protects mice from CHIKV-induced severe disease. (**A**) Scheme diagram of experimental design. In brief, mice were infected by subcutaneous (s.c.) injection in the left rear footpad with 1000 PFU of CHIKV-LR2006, followed by daily oral administration (p.o.) of nifuroxazide (50 mg/kg per day) from the day before infection to the third day post infection (−1 to 3 dpi). (**B**) Footpad swelling was monitored daily for 10 days. (n = 10) (**C**,**D**) Mice were euthanized at 2, 4, 6, and 8 dpi, and the ipsilateral (**C**) and the contralateral (D) feet were harvested for detection of viral RNA by RT-qPCR. (n = 6) (**E**). Sections of the ipsilateral feet of the mice euthanized at 4 dpi were prepared and stained with hematoxylin-eosin (HE). Scale bar, 50 μm. (**F**). Hematoxylin-eosin sections from panel E were scored blind for joint. Horizontal lines indicate the arithmetic mean. Mock: uninfected and DMSO-treated group; DMSO: infected and DMSO-treated group; Nifuroxazide: infected nifuroxazide-treated group. (n = 6) ** *p* < 0.01; *** *p* < 0.001 compared to DMSO control. NS: not significant.

We next followed whether nifuroxazide exhibited antiviral activity *in vivo* using a mouse model of CHIKV infection. Four-week-old C57BL/6 mice were inoculated subcutaneously (s.c.) in the footpad with CHIKV, treated by oral administration of nifuroxazide (50 mg/kg, Nifuroxazide group) or DMSO (DMSO group) daily from the day before infection to the third day post infection (−1–3 dpi), and monitored for footpad swelling daily for 10 days (Figure 5A). Conspicuous footpad swelling was observed in the CHIKV-infected mice (DMSO group) at 2 dpi and continued to grow to 6 dpi before relief (Figure 5B, the blue line). In comparison, repeated administration of nifuroxazide significantly relieved the swelling from 4 dpi to 8 dpi (Figure 5B, the red line).

To confirm the impact of nifuroxazide on CHIKV amplification *in vivo*, the viral RNA load was analyzed within both the ipsilateral and the contralateral feet at 2, 4, 6, and 8 dpi. We found that the level of CHIKV RNA was higher in the ipsilateral foot than in the contralateral foot of the infected mice, while it increased and reached the maximum at 4 dpi and then gradually decreased in both tissues (Figure 5C,D). As expected, the increasing of viral RNA copies at 4 dpi was dramatically lessened by repeated administration of nifuroxazide within both the ipsilateral and the contralateral feet (Figure 5D). The drug even worked at the early stage (2 dpi) of viral proliferation in the contralateral, but not the ipsilateral, foot. However, no obvious inhibitory effect was observed at 6 and 8 dpi, indicating that nifuroxazide may act mainly during the viral replication phase rather than the late clearance phase. Plaque assay was utilized to determine the infectious virions in the tissues on 2 and 4 pi. As expected, consistent results with that of viral RNA were observed in Figure S2. To further analyze the impact of nifuroxazide on CHIKV pathogenesis, mice were euthanized at 4 dpi, and inflammatory pathology was assessed within the ankle joints by HE-stained sections. As predicted, CHIKV infection induced widely inflammatory cell infiltration (the magnified boxes), interstitial hyperplasia (the magnified boxes), and muscle cell edema (the red arrow) within the joints, which were significantly improved by repeat treatment with nifuroxazide (Figure 5E). Blind histological analysis also indicated that infected mice under repeat treatment with nifuroxazide resulted in less damage within joints (Figure 5F). These results are consistent with the attenuation of footpad swelling and viral load.

## 4. Discussion

The increasing recognition of the burden imposed by arboviruses, associated with the limited therapeutic arsenal, has led to initiatives and strategies to research and develop new drugs for the treatment of arboviral diseases [30]. One of these strategies is drug repurposing, as a process of identifying new uses for approved or investigational drugs, which is considered a very effective strategy for drug discovery because it involves less time and cost to find a therapeutic agent than does the de novo drug discovery process [31]. Nifuroxazide, a well-known and often used antidiarrheal medicine, has been explored for its antibacterial and antiparasitic capabilities against bacteria and parasites [32]. To our knowledge, in this study, we firstly demonstrate that the antibacterial drug nifuroxazide exhibits antiviral properties against Chikungunya virus (CHIKV) *in vitro* and *in vivo*, providing novel prevention and therapy strategies for arboviruses.

*In vitro* experiments revealed concentration-dependent, detrimental effects of nifuroxazide on CHIKV, with peripheral cells being more susceptible to nifuroxazide than central nervous system cells. When the arbovirus enters the host through a mosquito bite, the virus replicates in keratinocytes, skin cells, and vein endothelial cells, and then arrives at and infects peripheral organs such as the liver and kidney through the blood and lymphoid circulation [4,5,6]. As shown in Figure 1 and Figure 2, nifuroxazide significantly inhibited CHIKV, YFV, TBEV, and WNV infection in multiple cell lines, such as Huh7 cells and HUVECs, revealing that nifuroxazide potentially protects against arbovirus infection in patients who experience disease progression or develop peripheral organ failure. Nevertheless, under CHIKV-/YFV-/TBEV-infection, nifuroxazide exhibited dull or weakened effectiveness in SY5Y cells, a model for studying viral invasion of neurons. On the one hand, there may be specific factors in SY5Y cells that interfere with the effect of nifuroxazide, such as competitive binding molecules for the target protein. On the other hand, it is possible that nifuroxazide targets a host protein that is critical for viral infection of peripheral cells, and within this protein in the neuronal cells, existing mutations lead to resistance to nifuroxazide but still support viral infection and replication. Besides, the antiviral mechanism of nifuroxazide in neurons cells may be different from that in peripheral cells under arbovirus infection. In any case, the exploration of the target protein will be the key to future research. In clinical practice, neurologic disease is not usually associated with CHIKV and YFV. Severe incapacitating arthralgia is the most prominent feature of acute CHIKV infection, and the enlargement, congestion, and edematous of the liver or kidney is the characteristic symptom of YFV infection [33,34]. It has been reported that over 50% of WNV-infected patients have neuroinvasive diseases that significantly increase the risk of death during acute hospitalization [35,36]. Nifuroxazide potentially inhibits WNV infection in SY5Y cells (Figure 2F), demonstrating that it might be effective at suppressing WNV invasion in the central nervous system, thus preventing potential neuroinvasive damage and decreasing patient mortality. Indeed, the inhibitory effect of nifuroxazide on WNV in SH-SY5Y cells at 25 and 50 μM may, due to the cytotoxicity, lead to death of the cells, as activation of cell death signals may also affect viral infection. Besides, for *in vitro* study, nifuroxazide was used from 2 h post infection (2 hpi) to 24 hpi, while the treatment of nifuroxazide was from the day before infection to the third day after infection *in vivo*. In other words, continuous dosing has a definite therapeutic effect *in vivo*. However, for clinical application, the dosing method of nifuroxazide for treatment or prevention still needs to be determined by further research.

Nifuroxazide presents as a potential anticancer agent depending on potent inhibition of the transcription factor signal transducer and activator of transcription STAT3 [37]. Subsequently, nifuroxazide was reported to induce cancer cell apoptosis and inhibit tumor growth [38]. Additionally, nifuroxazide was identified as a potent inhibitor of aldehyde dehydrogenase ALDH1 that selectively kills ALDH high-expression cancer-initiating cells [39]. Of note, in our study, nifuroxazide was involved in multiple stages of the CHIKV life cycles, especially the −2–10 hpi period, exhibiting prominent suppressive activity during the most therapeutic windows (Figure 3C). Moreover, the antiviral effect was apparent when it was maintained between 4 and 8 hpi, which suggested that nifuroxazide may disturb the package of CHIKV particles. We also demonstrated that assembly ratio decreased a few under treatment of high concentration nifuroxazide treatment (Figure 4B). Replication is a vital process which controls viral infection. Most of the FDA-approved antiviral drugs in clinical practice target viral key enzymes, such as the protease or polymerase [40]. Our study indicated that nifuroxazide suppresses the expression of CHIKV-LR2006 replicons and YFV-17D replicons. Additionally, it is worth considering that nifuroxazide may inhibit CHIKV through nsp4 or through other targets, which requires further study.

In order to evaluate the antiviral activity of nifuroxazide *in vivo*, a CHIKV arthritis mouse model was utilized. C57BL/6 mice are susceptible to CHIKV infection, which has been widely used in studies concerning the pathogenicity of CHIKV [41]. The results showed that nifuroxazide treatments effectively ameliorated footpad swelling and reduced the ipsilateral and the contralateral feet viral burdens at 2 dpi and 4 dpi. The results confirmed the antiviral activities of nifuroxazide *in vivo*. Future research will aim to evaluate the antiviral effects of nifuroxazide against different arboviruses *in vivo*. As an FDA-approved broad-spectrum antibacterial drug, the high tolerance and low toxicity of nifuroxazide was systematically evaluated [42]. Further studies on this mechanism ought to be carried out before nifuroxazide is applied as an antiviral agent in clinical settings.

In summary, FDA-approved nifuroxazide was uncovered as an effective anti-CHIKV agent both *in vitro* and *in vivo* via inhibiting viral replication, as well as a functional anti-flavivirus drug *in vitro*. Our results showed that nifuroxazide may be a promising broad-spectrum antiviral agent for use against arbovirus infections.

## Data Availability

The data that support the findings of this study are available from the corresponding author upon reasonable request.

## Supplementary Materials

The following supporting information can be downloaded at https://www.mdpi.com/article/10.3390/v16081322/s1, Figure S1. Schematic diagram of the replicon plasmids; Figure S2. Nifuroxazide treatment reduces infectious CHIKV titer in mice foot; Table S1. Selective index (SI) of nifuroxazide; Table S2. Information of the antibodies used for immunofluorescence; Table S3. Sequences of primer pairs specific to target genes.
