# Supplementary material for: Nifuroxazide Prevents Chikungunya Virus Infection Both In Vitro and In Vivo via Suppressing Viral Replication
Source: Viruses. 2024 Aug 19;16(8):1322. doi: 10.3390/v16081322 (PMC11360488; doi:10.3390/v16081322)
Supplement: Supplementary file 1 [file viruses-16-01322-s001.zip › Supplementary Information-revised-20240817.pdf]

Nifuroxazide prevents chikungunya virus infection both in vitro and in vivo via suppressing viral replication

Yangang Liu[1,2,†], Mingxiao Xu[3,†], Binghui Xia[1,2,†], Zhuoyue Qiao[4], Yanhua He[1,2], Yan Liu[1,2], Zhendong Pan[1,2], Congcong Zhang[1,2], Haoran Peng[1,2], Xuesong Liang[3], Ping Zhao[1,2], Hailin Tang[1,2,*], and Xu Zheng[1,2,*]

[1] Department of Microbiology, Faculty of Naval Medicine, Naval Medical University, 200433 Shanghai, China
[2] Key Laboratory of Biological Defense, Ministry of Education, Naval Medical University, 200433 Shanghai, China
[3] Department of Infection Diseases, First Affiliated Hospital of Navy Military Medical University, 200433 Shanghai, China
[4] Key Laboratory of Chemistry in Ethnic Medicinal Resources,State Ethnic Affairs Commission & Ministry of Education,Yunnan Minzu University, 650500 Kunming ,China
[*] Correspondence: hailint@163.com (H.T.); zhengxu87@nankai.edu.cn (X.Z.); Tel.: +86-021-81870990 (H.T.); +86-021-81870990 (X.Z.).
[†] These authors contributed equally to this work.

1. **Supplementary Figures**

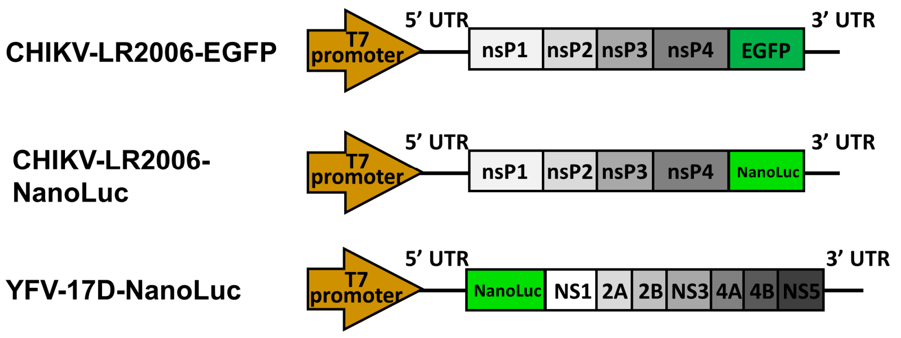

Figure S1. Schematic diagram of the replicon plasmids

T7 promoter is for *in vitro* transcription. nsP/NS: nonstructural protein. 2A: NS2A; 2B: NS2B; 4A: NS4A; 4B: NS4B. UTR: untranslated region. EGFP: enhanced green fluorescent protein.

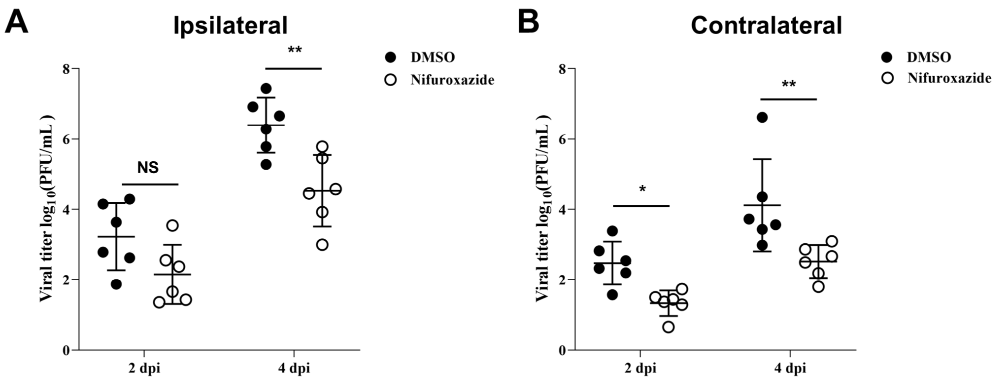

Figure S2. Nifuroxazide treatment reduces infectious CHIKV titer in mice foot.

Mice were infected by subcutaneously (sc.) injection in the left rear footpad with 100 PFU of CHIKV-LR2006 followed by daily oral administration (po.) of nifuroxazide (50 mg/kg·day^{-1}) from the day before infection to the third day post infection (-1 to 3 dpi.). (A, B) Mice were sacrificed at 2 and 4 dpi. and the ipsilateral (A) and the contralateral (B) feet were harvested for detection of infectious virions by plaque assay. DMSO: infected and DMSO-treated group; Nifuroxazide: infected and nifuroxazide-treated group. *$p < 0.05$,. **$p < 0.01$ compared to DMSO control. NS, not significant.

2. Supplementary Tables

Table S1. Selective index (SI) of nifuroxazide

Drug	Virus	Cell lines	IC50(μM)	CC50(μM)	SI
nifuroxazide	CHIKV	Huh 7	4.6	113.7	24.7
		SY5Y	110.1	186.5	1.7
		HUVEC	4.5	245.5	54.6
	YFV	Huh 7	1.3	113.7	87.5
		SY5Y	-	186.5	-
		HUVEC	9.6	245.5	25.6
	TBEV	Huh 7	19.6	113.7	5.8
		SY5Y	-	186.5	-
		HUVEC	8.1	245.5	30.3
	WNV	Huh 7	14.2	113.7	8.0
		SY5Y	32.6	186.5	5.7
		HUVEC	5.2	245.5	47.2

Table S2. Information of the antibodies used for immunofluorescence

Antibody	Species	Dilution	Supplier
anti-YFV	Rabbit	1:1000	prepared in the laboratory
anti-TBEV	Rabbit	1:1000	prepared in the laboratory
anti-WNV	Rabbit	1:1000	prepared in the laboratory
anti-CHIKV E1	Rabbit	1:500	BioFront Technologies, FL, USA
AF-488-conjugated anti-rabbit	Donkey	1:2000	Thermo Fisher Scientific, CA, USA

Table S3. Sequences of primer pairs specific to target genes

Target Gene	Sequences of qRT-PCR Primer Pair (5′-3′)	
Human-GAPDH	Forward Primer	TGGGCTACACTGAGCACCAG
	Reverse Primer	AAGTGGTCGTTGAGGGCAAT
Mouse-GAPDH	Forward Primer	TGGTGGACCTCATGGCCTACATGG
	Reverse Primer	TGAGGGAGATGCTCAGTGTTGGGG
CHIKV	Forward Primer	GGCAGTGGTCCCAGATAATTCAAG
	Reverse Primer	ACTGTCTAGATCCACCCCATACATG
